# Mild hyperthermia accelerates doxorubicin clearance from tumour-extravasated temperature-sensitive liposomes

**DOI:** 10.7150/ntno.61280

**Published:** 2022-01-01

**Authors:** Wafa' T. Al-Jamal, Kostas Kostarelos

**Affiliations:** 1 Nanomedicine Lab, UCL School of Pharmacy, University College London, Brunswick Square, London WC1N 1AX, United Kingdom.; 2 School of Pharmacy, Queen's University Belfast, United Kingdom.; 3 Faculty of Medical &Human Sciences and National Graphene Institute, University of Manchester, Manchester, United Kingdom.

**Keywords:** Intravascular release, interstitial release, hyperthermia, temperature-sensitive liposomes (TSL), doxorubicin, cancer

## Abstract

Mild hyperthermia (HT) (40-43 °C) has been combined with temperature-sensitive liposomes (TSL), offering on-demand drug release for increased drug bioavailability and reduced systemic toxicity. Different HT regimens have been applied to trigger liposome drug release in the blood vessels (intravascular) of heated tumours or following tumour extravasation (interstitial). The present study systematically assessed the *in vivo* doxorubicin (Dox) release and therapeutic efficacy of Dox-loaded TSL with different release profiles. Low temperature-sensitive liposomes (LTSL-Dox), traditional-temperature-sensitive liposomes (TTSL-Dox), and non-temperature-sensitive liposomes (NTSL-Dox) were combined with a single or two HT in different tumour models (murine melanoma B16F10 tumour and human breast MDA-MB-435). The efficacy of each treatment was assessed by monitor tumour growth and mice survival. The level of Dox in tumour tissues was quantified using ^14^C-Dox and liquid scintillation while Dox release was assessed using live imaging and confocal laser scanning microscopy. Applying a second HT to release Dox from extravasated TTSL-Dox was not therapeutically superior to single HT application due to Dox clearance from the extravasated TTSL-Dox. Our findings revealed that enhanced blood perfusion in heated tumours during the second water bath HT could be seen as a hurdle for TTSL-Dox's anticancer efficacy, where the systemic toxicity of the redistributed Dox from the tumour tissues could be potentiated.

## Introduction

Mild localised hyperthermia (HT) is known to increase blood flow and interstitial microconvection transports of small and macromolecules 1-3. It can also increase the gaps in endothelial linings, thus facilitate nanoparticle extravasation and subsequent penetration in the tumour extracellular space 2, 3. Furthermore, clinically-attainable mild HT (40-43 °C) has been used as an adjuvant to chemotherapy and radiotherapy to enhance the therapeutic efficacy of existing cancer treatments 1, 4 by improving tissue oxygenation, sensitizing cancer cells to cytotoxics, and inhibiting DNA repair 5-7.

HT has been combined with temperature-sensitive liposomes (TSL) to trigger drug release and overcome low drug bioavailability. Based on the HT regimen and the TSL characteristics, the release profile could be altered to obtain intravascular or interstitial drug release. In the intravascular drug release approach, HT is applied shortly before or after liposomes injection, which triggers ultrafast Dox-release from circulating TSL in the tumour vasculature 8, 9. On the contrary, interstitial drug release, HT is combined with serum-stable, long-circulating TSL with an intermediate release profile, where HT is applied after several hours to release the drug from extravasated liposomes in the tumour tissue. ThermoDox^®^ is a low-phase transition TSL formulation that exhibits an ultrafast release kinetic profile suitable for intravascular drug release. It is clinically infused in patients over 30 min before applying HT 10.

A two-step HT approach has been proposed to enhance liposomal delivery and release in tumours, where it could be difficult to apply HT precisely 11. This approach relies on applying HT prior to liposomal injection, aiming to increase blood flow and blood vessel permeability in tumour regions, which ultimately leads to significant improvement in tumour accumulation of liposomes, followed by a second HT to release the encapsulated drug from the extravasated liposomes at the tumour interstitium. Lately, there has been growing interest in applying this approach to enhance the therapeutic efficacy of serum stable, long-circulating TSL with an intermediate release profile 11, 12. Several *in vivo* studies have reported the lower therapeutic efficacy of such approach compared to mice injected with fast-releasing TSL-Dox formulations combined with a single HT treatment. Despite the fact that these studies explored different TSL formulations and two-step HT approaches 11-13, the majority of these reports relied on assessing the therapeutic efficacy of this HT regimen by delaying tumour growth, but with limited quantification of Dox (released or encapsulated) in the tumour tissues.

Enhanced tumour blood perfusion during the second HT application could lead to Dox clearance, which counteracts the efficacy of TSL-Dox with an intermediate release profile. To investigate this hypothesis, we advanced our previous comprehensive pharmacokinetics study 14 by systematically evaluating Dox release *in vivo*, and the therapeutic efficacy of lysolipid-containing temperature-sensitive liposomes (LTSL-Dox), traditional-temperature-sensitive liposomes (TTSL-Dox), and non-temperature-sensitive liposomes (NTSL-Dox) combined with a single or two HT in two tumour models (murine melanoma B16F10 tumour and human breast MDA-MB-435). Our results have demonstrated a significant Dox clearance from tumours injected with TTSL-Dox and subjected to two HT (60 min), resulting in therapeutic efficacy similar to mice treated with TTSL-Dox and a single session of HT. This phenomenon should be considered in cancer therapy since second HT, using water bath or other modalities, could promote systemic toxicity from the redistributed Dox to the blood circulation.

## Materials and Methods

### Materials

Dipalmitoylphosphatidylcholine (DPPC); hydrogenated soy phosphatidylcholine (HSPC); 1,2-distearoyl-sn-glycero-3-phosphoethanolamine-N-[methoxy(polyethylene glycol)-2000] (ammonium salt) (DSPE-PEG2000) were kind gifts from Lipoid GmbH (Germany). 1-stearoyl-2-hydroxy-sn-glycero-3-phosphocholine (MSPC) were purchased from Avanti Polar lipid (Alabama, USA). Cholesterol, isopropanol, chloroform, methanol, 4-(2-Hydroxyethyl)piperazine-1-ethanesulfonic acid (HEPES), and doxorubicin hydrochloride (Dox) were purchased from Sigma (UK). [^14^C] Doxorubicin hydrochloride (^14^C-Dox) were purchased from PerkinElmer (UK). All chemical substances and solvents were used without further purification.

### Preparation of Dox-loaded liposomes

Phospholipids were dissolved in chloroform: methanol (4:1 v/v) mixture. Multilamellar vesicles (MLV) were prepared by evaporating the organic solvent under vacuum for 30 min at 40 °C using a rotovaporator (BÜCHI, Switzerland), then flushed with N_2_ stream to remove any residual traces of organic solvent. The dried lipid films of LTSL [DPPC:MSPC:DSPE-PEG_2000_ (86:10:4)]; TTSL [(DPPC:HSPC:Chol:DSPE-PEG_2000_(50:25:15:3)]; and NTSL [(HSPC:Chol:DSPE-PEG_2000_ (75:50:3)] were hydrated with ammonium sulphate buffer pH 5.4 (24 0mM (NH_4_)_2_SO_4_) to achieve a final lipid concentration of 25 mM. Small unilamellar vesicles (SUV) were prepared by extrusion at 60 °C through 800 nm, 200 nm, and 100 nm polycarbonate filters 5 times each, followed by 10 times extrusion through 80 nm membranes using a mini-Extruder (Avanti Polar Lipids, USA), then flushed with N_2_ and stored in the fridge to anneal overnight. Liposome size and surface charge were measured by using Zetasizer Nano ZS (Malvern, UK, He-Ne laser). Dox was loaded into liposomes using the pH-gradient method. Briefly, liposome external buffer was exchanged using PD-10 Sephadex G-25 (GE Healthcare Life Sciences, UK) equilibrated with HBS pH 7.4 (20 mM HEPES, 150 mM NaCl). Dox hydrochloride (5 mg/ mL) was added to the desalted liposomes suspensions at 20:1 lipid:Dox mass ratio in respect to the original total lipid concentration. Subsequently, samples were incubated for 90 min at 37 °C in the case of LTSL, 1 hr at 60 °C for NTSL and overnight at 39 °C for TTSL. For *in vivo* studies, radiolabelled liposomes were prepared by loading them with ^14^C-Dox. Liposomes were loaded with Dox solution spiked with ^14^C-Dox to maintain the lipid:Dox mass ratio at 20:1. Unencapsulated Dox and ^14^C-Dox were removed as described above. Liposomes were concentrated using Vivaspin 300,000 MWCO (Vivascience, Sartorus, Germany). Dox encapsulation efficiency was determined, as previously reported 14.

### Characterisation of Dox-loaded liposomes

Liposome size and surface charge were measured by using Zetasizer Nano ZS (Malvern, UK, He-Ne laser), as previously described (14). In order to determine the phase transition temperatures of the liposomes, 20 μL of multilamellar vesicles (MLV) suspensions was placed in T zero hermetic aluminum pans sealed with lids. Samples were then thermally scanned from 30 °C to 60 °C at 1 °C/min heating rate using differential scanning calorimetry (DSC) (Q2000 differential scanning calorimeter, TA Instruments, USA).

### Animal model

All animal experiments were performed in compliance with the UK Home Office Code of Practice for the Housing and Care of Animals used in Scientific Procedures. 6-8 week-old female C57BL6 mice (15-20 g) (Harlan UK Limited, UK) were caged in groups of 5 with free access to water. 5-6 week-old female Athymic nude mice (16-20g) (Harlan UK Limited, UK) were caged in individually vented cages (IVC; Allentown, USA) in groups of five with free access to food and water. A temperature of 19-22 ºC was maintained, with a relative humidity of 45-65 %, and a 12-hr light/dark cycle. All animals were acclimatised for 7 days before the experiment. Athymic nude mice were kept on Teklad 2019 purified diets (Harlan UK Limited, UK) since arrival, which was essential for MDA-MB-435 tumour growth and fluorescence live imaging. To establish a syngeneic (B16F10 melanoma, ATCC, USA) tumour model, mice were inoculated subcutaneously on the hind leg with 0.25 x 10^6^ B16F10 melanoma cells in a volume of 20 μL PBS using a 26 G needle. To establish MDA-MB-435 xenografts, athymic nude mice were inoculated subcutaneously on the right and left flanks with 1 × 10^7^ (150 μl) MDA-MB-435 breast cancer cells suspended in serum-free DMEM media. MDA-MB-435 cells were a kind gift from Dr John Maher, King's College London. The tumour volume was estimated by measuring three orthogonal diameters (a, b, and c) with callipers; the volume was calculated as (a× b× c) × 0.5 mm^3^. Biodistribution studies were performed when the tumour volume reached 250-500 mm^3^. All therapy experiments were carried out when B16F10 tumours reached a volume of 50-60 mm^3^ and MDA-MB-435 xenograft volume of 100-150 mm^3^.

### Hyperthermia and biodistribution of Dox-loaded TSL *in vivo*

Mice were anaesthetised by inhalation of isoflurane and injected via the tail vein with 200 μL of the liposomes suspension in HBS at 5 mg/kg of Dox (approximately 100 µg Dox/mouse). Radio-labeled liposomes were injected at a dose of 0.25-0.3 µCi ^14^C/mouse. For a single HT treatment, local HT was applied immediately after liposome injection and maintained for 60 min, as described previously (14). For two HT, a second HT (60 min) was applied 24 hr after the first HT application. For Dox clearance studies, the second HT duration was varied between 15-60 min. Anaesthetised mice were then placed on a Polyvinyl chloride (PVC) stage containing holes, which was placed over water bath set up at 43.5 °C (Grant, Germany) so the tumour-bearing legs were immersed completely in 42.5 °C water bath (water temperature as measured by a thermocouple). During HT, animals were anesthetised by inhalation of isoflurane and the body temperature of the mice was monitored with a rectal thermocouple. A fan and a heating pad were used to maintain the body temperature at 36-37 °C. Mice were culled 2 hr after the first or second HT to allow Dox cell uptake, or 24 hr after the first HT. Mice were culled by cervical dislocation, and organs and tumours were excised and analysed for radioactivity, as described below.

### Radioactivity Measurements

Radioactivity measurements were performed as previously described 14. Tumour samples were transferred to 20 mL scintillation vials and solubilised with 1 mL of Soluene-350 tissue solubiliser (PerkinElmer, UK), and shaken overnight at 55 °C. Before adding the scintillation cocktail samples were decolorized by adding 0.3 mL of 30% H_2_O_2_ and isopropanol as an antifoaming agent. Samples were shaken at 55 °C for 1-3 hr to expel H_2_O_2_ before adding the scintillation cocktail. Samples were then mixed with 20 mL of Optiphase “Safe” scintillation cocktail (Fisher Scientific, UK) acidified with 0.7% (v/v) glacial acetic acid to eliminate any chemiluminescene. ^14^C radioactivity was quantified simultaneously for each sample using the LS6500 multipurpose scintillation counter (Beckman, USA). The results were represented as the percentage of the injected dose (% ID) per gram tissue.

### Fluorescence imaging

Mice were anesthetized using isofluorane and injected intravenously with Dox-loaded liposomes at 5 mg /kg Dox (150 µg Dox) or intratumourally (20 ug Dox) and HT was applied immediately, as described above, and followed by IVIS imaging. For intratumoural injection, the needle was inserted in the longitudinal direction from the tumour edge into the center of the tumour, 40 µL of the dispersion was administered slowly over 1 min, and the needle was left in the tumour for another 5 min to prevent sample leakage. Due to the high fluorescent background of C57BL6 mice, experiments were carried out only in athymic nude mice.

#### *In vivo* optical imaging

Live animal fluorescence optical imaging was used to monitor Dox release *in vivo* using the IVIS® Lumina II *In vivo* Imaging System (Caliper Life Sciences Corp., Alameda, CA). Images were acquired and analysed using Living Image software 4.0 (Caliper Life Sciences Corp.). All images were acquired using the following settings: binning= 4, exposure time= 3-20 sec, a field of view= 12.5 or 24, f-stop=2 and filters with an excitation of 500 nm and emission of 575-650 nm. Images were corrected by subtraction from background images at 430 nm excitation wavelength and GFP emission filter. Dox fluorescent intensity at the tumour site was quantified by drawing a region of interest (ROI) that covers the tumour area. Data are displayed in the unitless value of efficiency and represent the ratio of light emitted to light incident.

#### Tissue analysis

Mice were culled by cervical dislocation, and tumours were harvested and stored at -80 °C prior to frozen sectioning. Frozen tumours were embedded into OCT and sectioned using the cryostat (Leica Microsystems, CM3050S) at -18 °C into 10 µm thick sections. The sections were mounted on superfrost slides and left to dry at room temperature for 15-30 min. For tumour visualisation, the sections were fixed for 3 min in cold acetone at -20 °C, rinsed with PBS for 15 min at room temperature. The sections were mounted in a suitable medium containing anti-fading agent (Vectashield mounting medium, Vector Laboratories) and visualized using 488 nm laser excitation source, 534 nm output filter, under X63 oil immersion lens using a Zeiss LSM710 (Carl Zeiss Inc.).

### Therapeutic efficacy and survival studies

B16F10/C57BL6 tumour-bearing and MDA-MB-435 tumour-bearing mice were randomly divided into groups. Treatment was started when the tumour reached a volume of 50-60 mm^3^ (B16F10) or 100-150 mm^3^ (MDA-MB-435). All groups had the same mean tumour volume and standard deviation to reduce the variations during therapy. Groups were injected intravenously with 5 mg Dox/kg of LTSL-Dox, TTSL-Dox, and NTSL-Dox. The control group was injected with saline. For all groups local HT was applied immediately after injection for 60 min as a single treatment or in combination with a second HT treatment (60 min) 24 hr after the first HT application. Tumour volumes were monitored three times a week. Mice were culled if the tumour dimensions exceeded 10x15 mm. At the end of the experiment, organs and tumours were immediately fixed in 10% neutral buffer formalin. These tissues were then paraffin-embedded and sectioned for haematoxylin and eosin stains (H&E) and Masson's trichrome (to detect fibrosis) according to standard histological protocols at the Royal Veterinary College (London, UK). Survival time was recorded in days after tumour inoculation and graphed using GraphPad Prism software.

### Statistical analysis

For all tumour release and tumour growth delay studies, significant differences were examined using one-way ANOVA followed by Bonferroni post-test. *p* < 0.05 was considered statistically significant in all studies (* *p* < 0.05, ** *p* < 0.01, *** *p* < 0.001). The survival curves between different groups were compared using the Kaplan-Meier method by GraphPad Prism V5.0. The statistical analysis was performed using Log-rank (Mantel-Cox) test and the p values were later adjusted by Bonferroni method.

## Results

### Effect of mild HT on TSL-Dox tumour uptake

**Figure 1:**
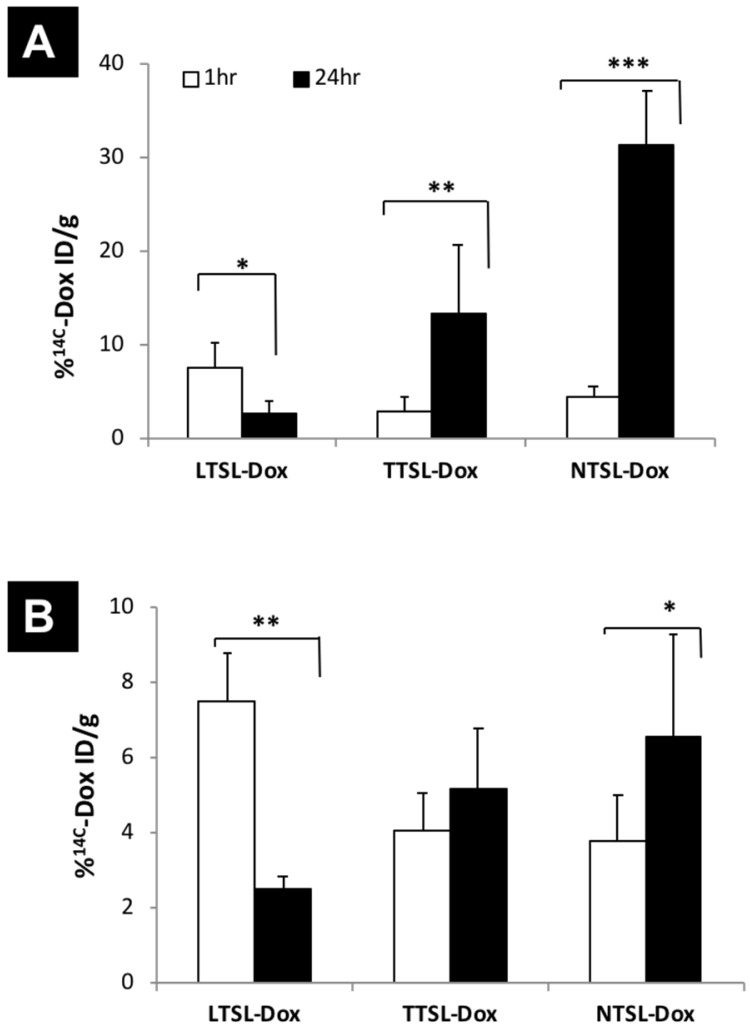
** Tumour accumulation of TSL-Dox in tumour-bearing mice in combination with mild localised HT.** ^14^C-Dox-loaded TSL (LTSL-Dox, TTSL-Dox and NTSL-Dox) were injected intravenously in **(A)** murine melanoma B16F10-tumour bearing mice and **(B)** human breast MDA-MB-435-xenograft bearing athymic nude mice. Mild localized HT (60 min) was applied immediately after injection, tumours were isolated 1 hr or 24 post-HT. 14C-Dox level in tumours was determined using liquid scintillation counter and results were expressed as % ID/g tissue. Data are expressed as mean ± S.D (n=4-5). Statistical analysis was performed using one-way ANOVA followed by Bonferroni post-test. * denotes comparison between control and the treatments (* p < 0.05, ** p < 0.01, *** p < 0.001).

^14^C-Dox-loaded liposomes (LTSL-Dox, TTSL-Dox and NTSL-Dox) were prepared, as previously described. All formulations had comparable size, surface charge and Dox loading efficiency (**Table 1**). TTSL-Dox and TTSL-Dox exhibited low (41.5 ºC) and intermediate (44.2 ºC) phase transition temperature, respectively, compared to NTSL-Dox (**Table 1**), which agrees with our previous findings (14). ^14^C-Dox tumour uptake was assessed in a highly vascularised B16F10 tumour model and in less vascularised human breast MDA-MB-435 xenograft model. In agreement with our previous study (14), injecting liposomes combined with mild localised HT, LTSL-Dox showed the highest Dox level in the B16F10 tumours (7% ID/g) compared to TTSL-Dox and NTSL-Dox (2-4% ID/g tumour). Following 24 hr of injection, Dox levels in the tumours injected with LTSL-Dox showed 50% reduction due to Dox wash-out from the tumour following the intravascular Dox release 14. On the other hand, a four- to six-fold increase in Dox level was observed in the B16F10-tumour bearing mice injected with serum-stable and long circulating, TTSL-Dox and NTSL-Dox, respectively (**Figure 1A**). Consistently, the intravascular release showed the highest Dox level in the MDA-MB-435 model (7% ID/g tissue) before a significant reduction was observed 24 hr following the HT application. The lower vascularisation/permeability of this model was confirmed by the lower tumour accumulation (5-6% ID/g) of TTSL-Dox and NTSL-Dox after 24 hr of HT (**Figure 1B**).

### Effect of single and two HT sessions on the therapeutic efficacy of TSL-Dox in the murine melanoma B16F10 tumour model

**Figure 2:**
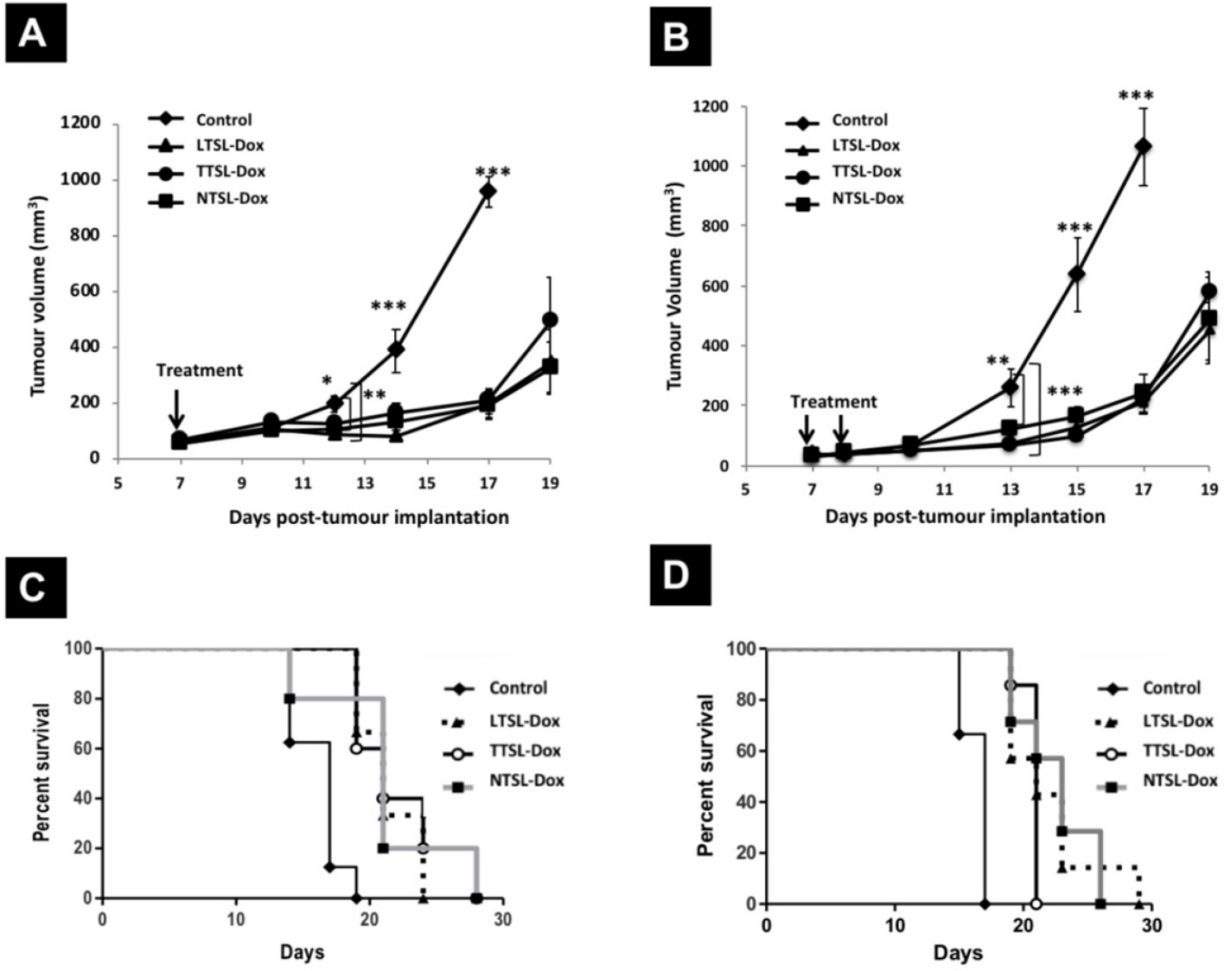
** Therapeutic efficacy of TSL-Dox and HT in B16F10 tumour-bearing mice.** Growth curves B16F10 tumours treated with TSL-Dox and **(A)** one HT or **(B)** two HT. Survival curves of B16F10 tumour-bearing mice treated with TSL-Dox and **(C)** one HT or **(D)** two HT. B16F10 cells (2.5x10^5^) were injected subcutaneously in the left leg, and therapy started on day 7 with tumour size of 50-60 mm^3^. Animals were injected intravenously with 5 mg Dox/kg of liposome-Dox formulations (LTSL-Dox, TTSL-Dox, NTSL-Dox) followed by 60 min HT by immersing the tumour-bearing leg into 43 °C water bath. HT was applied immediately after injection, and mice were subjected to second HT (60 min) 24 hr post the first HT. Control animals were treated with HT with no Dox injection. Each group (n=5-6, error bars: ± S.E.M). For the tumour growth study, statistical analysis was performed using one-way ANOVA followed by Bonferroni post-test. * denotes comparison between control and the treatments (* p < 0.05, ** p < 0.01, *** p < 0.001).

In this study, we investigated the therapeutic efficacy of TSL-Dox with different serum stability and release profiles in the B16F10 tumour model, using one or two HT protocols. B16F10 tumour-bearing mice were injected intravenously with LTSL-Dox, TTSL-Dox, or NTSL-Dox, then tumour-bearing legs were immediately immersed for 60 min in 43 ºC water bath. For the two HT treatment, the injected mice were subjected to a second 60 min HT, 24 hr post the first HT treatment, to trigger Dox release interstitially. Control mice were also treated with HT to account for any effects of HT alone on the tumour growth. All tumours treated with liposome-Dox formulations in combination with mild localised HT were significantly smaller compared to the control group throughout the whole study (**Figure 2A**). In addition, liposome-Dox treated groups significantly showed a longer survival (21 days) compared to the control group (17 days) (**Figure 2C, Table 2**). LTSL-Dox combined with HT was the most effective in retaining a smaller tumour volume (**Figure 2A, triangles**). This effect from LTSL-Dox injection was transient since there was no statistical significance between all treated groups by day 17. As expected, despite the higher levels of Dox in the tumours injected with TTSL-Dox and NTSL-Dox after 24 hr post-HT (**Figure 1A**), the therapeutic efficacy and survival of those animals were not dramatically improved (**Figure 2C**), presumably due to the low bioavailability of Dox in the tumour tissue. No toxicity was observed in any of the treated groups up to three weeks post-injection. This was confirmed by histological examination using H&E (**Figure S1**) and Masson's Trichome staining (**Figure S2**), where only some macrophages infiltration was observed in the liver of the treated groups (**Figure S1**).

In order to enhance the therapeutic efficacy of TSL-Dox formulations in the highly vascularised, fast-growing B16F10 tumour model, a second HT treatment was applied 24 hr post-injection the first HT to trigger Dox release from TTSL at the tumour interistium. Surprisingly, no differences were observed in tumour growth in all mice injected with liposome-Dox formulations combined with the two HT protocols (**Figure 2B**). Our results also showed no significant increase in the median survival between the treated groups using one or two HT treatments (21-23 days *vs.* 17 days for the control mice) (**Figure 2D, Table 2**). This was further confirmed by examining tumour sections from the mice treated with single or two HT sessions, where similar tumour damage was observed in all treated groups (**Figure S3 & S4**).

### Effect of single and two HT sessions on the therapeutic efficacy of TSL-Dox in the human breast MDA-MB-435 xenograft model

**Figure 3:**
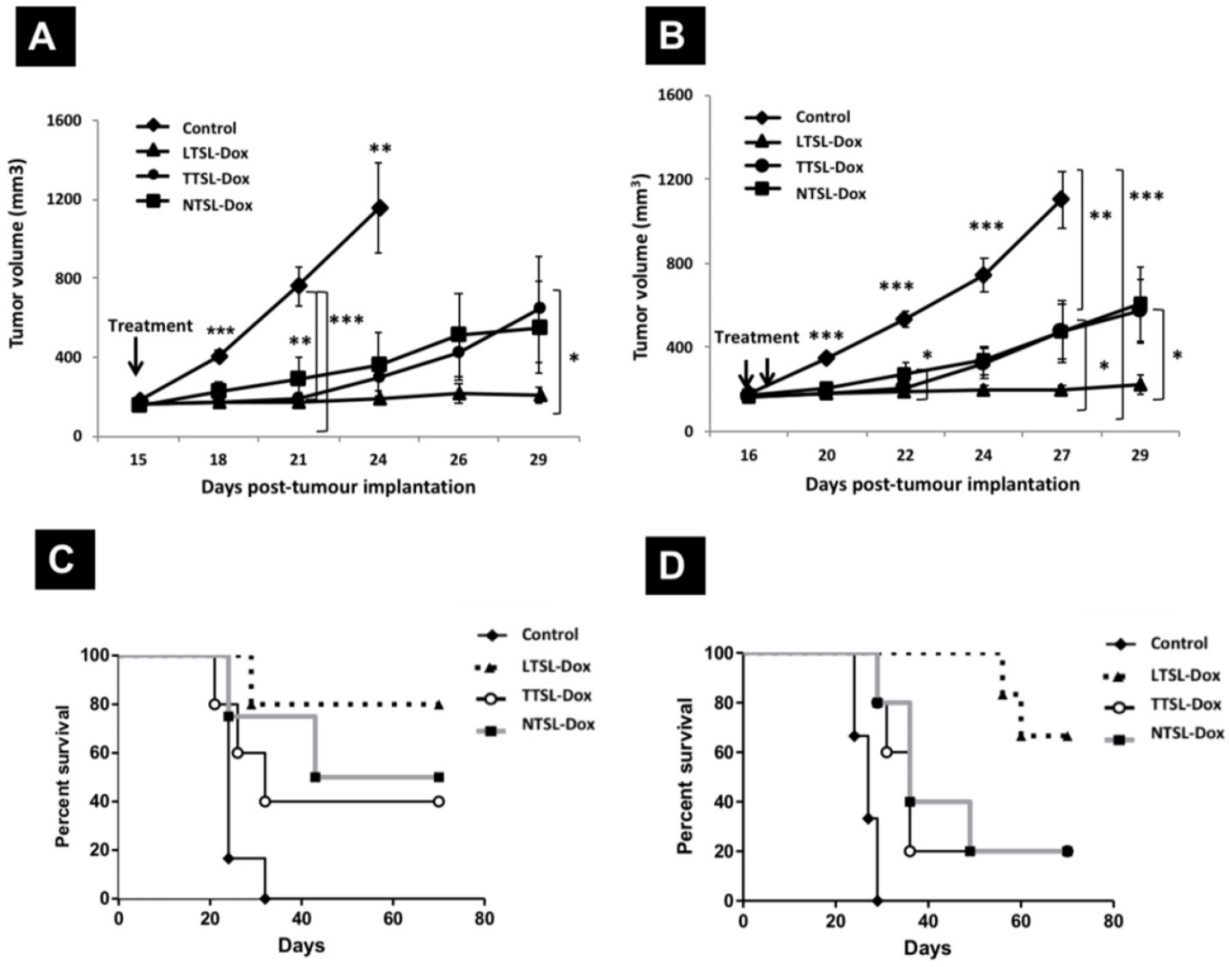
** Therapeutic efficacy of TSL-Dox and HT in MDA-MB-435 xenograft- bearing mice.** Growth curves MDA-MB-435-xenografts treated with TSL and **(A)** a single HT or **(B)** two HT. MDA-MB-435 cells (1 x 10^7^) were injected subcutaneously in the left leg and therapy started on day 15 or 16 with tumour size of 100-150 mm^3^. Animals were injected intravenously with 5 mg Dox/kg of liposome-Dox formulations (LTSL-Dox, TTSL-Dox, NTSL-Dox) followed by 60 min HT by immersing the tumour-bearing leg into 43 °C water bath. HT was applied immediately after injection, and mice were subjected to second HT (60 min) 24 hr post the first HT. Control animals were treated with HT with no Dox injection. Each group (n=5-6, error bars: ± S.E.M). For the tumour growth study, statistical analysis was performed using one-way ANOVA followed by Bonferroni post-test. * Denotes comparison between control and the treatments (* p < 0.05, ** p < 0.01, *** p < 0.001).

To assess the effect of tumour vascularity on the efficacy of TSL-Dox combined with a single or two HT protocol, we repeated the same experiments described above in less vascularised MDA-MB-435 tumour model. **Figure 3** depicts the tumour growth curves of MDA-435 tumours injected with TSL-Dox formulations and subjected to single HT immediately after injection, or to second HT following liposomes extravasation (24 hr post-injection). Contrary to the B16F10 tumour model, MDA-MB-435-xenograft-bearing mice injected with LTSL-Dox liposomes combined with single HT showed the slowest tumour growth compared to mice treated with TTSL-Dox or NTSL-Dox, where the latter had similar tumour growth curves (**Figure 3A**). Consistent with the B16F10 tumour results, second HT treatment, 24 hr post the first HT, did not affect tumour growth in all treated groups (**Figure 3B**). Furthermore, mice survival was similar to the ones treated with single HT (**Figure 3C & 3D**, **Table 2**), except for shorter median survival, observed in NTSL-Dox mice, which requires further investigation. As expected, the longest median survival was observed in mice treated with LTSL-Dox, where the median survival could not be determined since 70-80% of the mice were still alive at the end of both studies (70 days).

### Effect of single and two mild HT sessions on Dox release from TSL *in vivo*

In order to understand the therapeutic efficacy results obtained above, we decided to investigate the effect of single and two mild HT on Dox release *in vivo*. For this experiment, Dox-loaded liposomes (LTSL-Dox, TTSL-Dox, and NTSL-Dox) were injected intravenously in B16F10-tumour bearing C57BL6 mice and MDA-MB-435 xenograft-bearing athymic mice, and HT was applied immediately to trigger Dox release intravascularly, or 24 hr after the first HT, to trigger Dox release interstitially. The MDA-MB-435 xenograft-bearing mice were imaged immediately after the first HT, 24 hr post-HT, and after the second HT treatment (2H), using IVIS *In vivo* imaging of C57BL6, which could not be performed due to Dox quenching in the black mice. Tumours from both models were collected at these time points, snap frozen, cryo-sectioned, and examined under a confocal laser scanning microscope.

**Figure 4:**
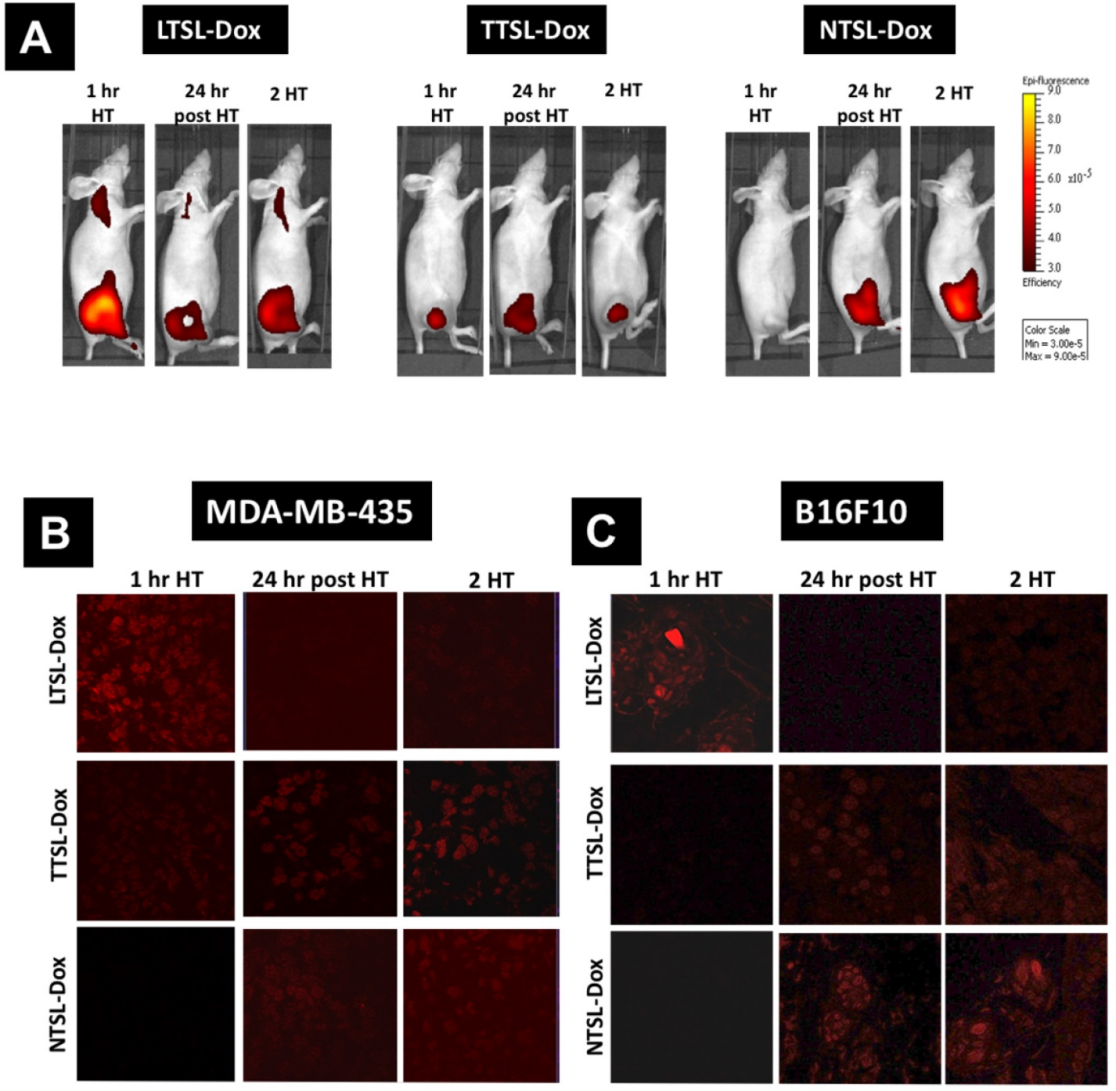
** TSL-Dox release *in vivo.*** TSL-Dox formulations were injected intravenously at 5 mg/kg Dox in tumour-bearing mice, and mild localised HT (60 min) was applied immediately after injection or 24 hr post the first HT. **(A)** Live imaging of Dox release in human breast MDA-MB-435 xenograft-bearing athymic nude mice, immediately after HT, 24 hr post-HT and after the second HT treatment (2 HT). At the end of the experiment, tumours were isolated from **(B)** MDA-MB-435 xenograft-bearing athymic nude mice and **(C)** murine melanoma B16F10-tumour bearing mice, fixed, cryo-sectioned, and visualised under the confocal laser scanning microscope.

**Figure 4 and Figure S5** depict the *in vivo* Dox release from the different TSL formulations combined with mild localised HT. MDA-MB-435 xenograft-bearing mice injected with LTSL-Dox showed the highest Dox release immediately after the first HT, however, 50% reduction in the fluorescence intensity was observed 24 hr later. Some Dox was also released in the heated leg of mice injected with LTSL-Dox, since the whole leg was immersed in the water bath. However, this undesirable release could be minimized by using more focused HT approaches, such as high intensity focused ultrasound (HIFU) 15, 16. As anticipated, applying the second HT protocol did not dramatically affect Dox intensity in tumours (**Figure 4A, left & Figure S5**). Mice injected with TTSL-Dox, had lower Dox signals after the first HT due to the intermediate release profile of this formulation, but exhibited a continuous increase in Dox fluorescence intensity, reaching the highest level 24 hr post the first HT (**Figure 4A, middle & Figure S5**). Surprisingly, rather than an increase in Dox fluorescence, which indicates Dox release, a reduction in Dox signals was observed after applying the second HT. NTSL-Dox, which was used as a negative control, did not show any Dox release in combination with HT, but Dox release was observed after 24 hr, and applying a second HT did not dramatically affect Dox signals (**Figure 4A, right & Figure S5**).

*Ex-vivo* tumour sections examination after the first HT revealed that the intravascular Dox release from LTSL-Dox resulted in the highest fluorescence intensity in the tumour sections (**Figure 4B & 4C**). Lower signals were observed in the sections of mice injected with TTSL-Dox (**Figure 4B & 4C**). No Dox release was observed with NTSL-Dox. 24 hr post the first HT, LTSL-Dox sections exhibited lower fluorescence signals, which agreed with Dox wash-out from the tumour (**Figure 1& 4**). On the other hand, higher intensities were observed in the TTSL-Dox and NTSL-Dox treated sections. This could be explained by passive Dox release from the extravasated liposomes in the tumour interstitium. Similar to *in vivo* imaging (**Figure 4A**), all sections treated with second HT, demonstrated similar levels of fluorescence to the sections prior to the second HT. No significant differences were observed between the two tumour models (**Figure 4B & 4C**).

### Effect of single and two HT sessions on Dox levels in tumour tissues

**Figure 5:**
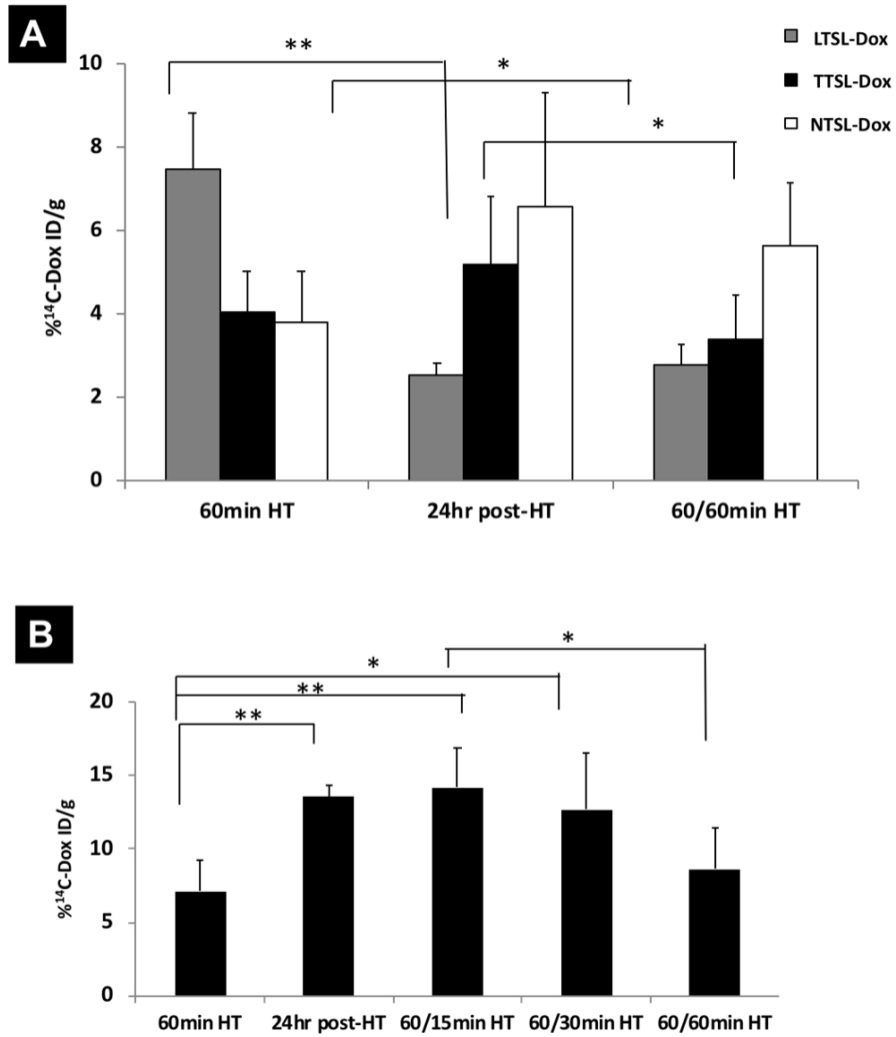
**Dox levels in tumour tissues in mice treated with TTSL-Dox and two mild localised HT. (A)** ^14^C-Dox-loaded TSL (LTSL-Dox, TTSL-Dox, or NTSL-Dox) were injected intravenously in human breast MDA-MB-435 xenograft-bearing athymic nude mice. Tumours were isolated immediately after 60 min HT, 24 hr post-HT, and after applying a second 60 min HT (60/60 min). **(B)** Murine melanoma B16F10-tumour bearing mice were injected with ^14^C-Dox-loaded TTSL, and tumours were isolated immediately after 60 min HT, 24 hr post-HT, and after applying second HT, which varied between 15-60 min (60/15 min, 60/30 min and 60/60 min). ^14^C-Dox level in tumours was determined using liquid scintillation counter, and results were expressed as %ID/g tissue. Data are expressed as mean ± S.D (n=4-5). Statistical analysis was performed using one-way ANOVA followed by Bonferroni post-test.* denotes comparison between control and the treatments (* p < 0.05, ** p < 0.01).

Following observing the reduction in Dox fluorescence in mice injected with TTSL-Dox combined with two HT sessions, we investigated the possibility of Dox clearance due to increased tumour blood flow. In this experiment, we quantitatively measured the total Dox the in MDA-MB-435 tumours following one or two HT sessions, using ^14^C-Dox and liquid scintillation counting. In agreement with our fluorescence imaging (**Figure 4**), applying the second HT to the mice injected with ^14^C-Dox loaded LTSL and NTSL did not tremendously affect ^14^C-Dox clearance since no significant differences were observed in the tumours before and after the second HT. This indicates that the increased flood flow neither washed the intracellular Dox, which was released from LTSL-Dox (**Figure 5A, grey bars**), nor the stable NTSL-Dox (**Figure 5A, white bars**). However, a significant reduction in ^14^C-Dox level was observed in the mice injected with ^14^C-Dox-loaded TTSL and subjected to second HT for 60 min (**Figure 5A, black bars**). This indicates slow release of ^14^C-Dox from the extravasated TTSL-Dox, which agrees with the release profile of these liposomes *in vitro*, where a complete release was achieved in 15-20 min (14). We speculated that the increased blood flow in the tumour washed the released ^14^C-Dox, which agrees with our previous studies 14. A similar experiment was repeated with ^14^C-Dox-loaded TTSL in the highly vascularised B16F10 tumour model. In this experiment, we also varied the duration of the second HT between 15-60 min. Consistently, our results confirmed more pronounced reduction in Dox level after 60 min HT, where the ^14^C-Dox level decreased by 50% (from 14% ID/g to 7% ID/g tumour) (**Figure 5B**). Shorter HT (15-30 min) did not affect the level of Dox in B16F10 tumours.

### Effect of blood flow on Dox tumour level following intratumoural administration of TSL-Dox

**Figure 6:**
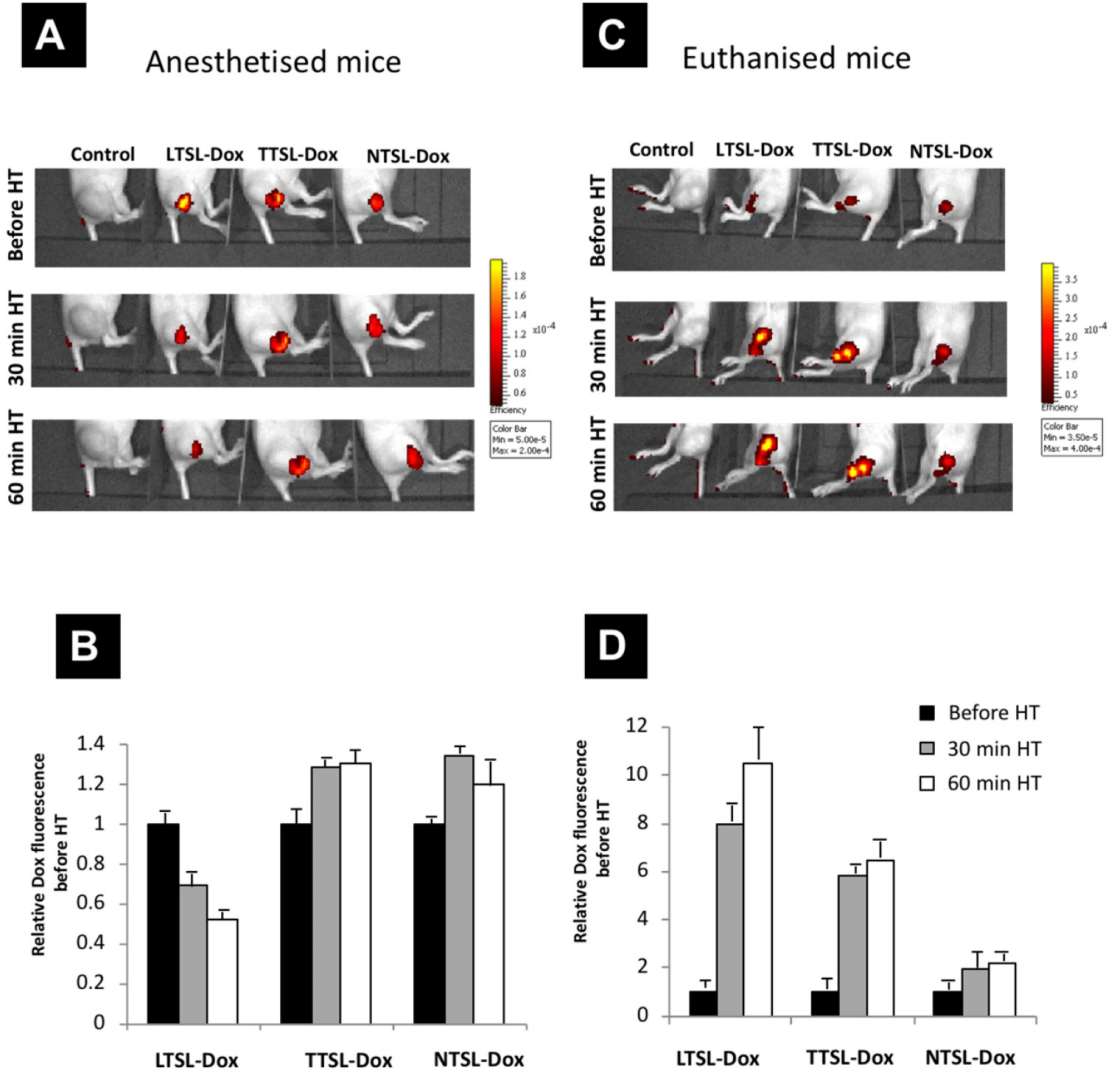
** TSL-Dox release *in vivo* following intra-tumoural injection*.*** TSL-Dox formulations (LTSL-Dox, TTSL-Dox and NTSL-dox) were injected intratumorally in **(A)** anaesthetised or **(C)** euthanized mice. Tumour-bearing legs were immediately immersed in a 43 ºC water bath for 30-60 min, and Dox release was imaged in mice using the IVIS imager. **(B and D)** are relative Dox fluorescence intensities to zero time point (before HT application) injected in anaesthetised and euthanised mice, respectively. Data are expressed as mean ± S.D (n=3).

To test the effect of accelerated blood flow caused by mild HT on Dox release and clearance from tumour tissues, TSL-Dox formulations (LTSL-Dox, TTSL-Dox, and NTSL-Dox) were injected intratumorally in euthanised mice with an absent tumour blood flow, or in anaesthetised mice with normal blood flow that responses to mild HT. Tumour-bearing legs were immediately immersed in a 43 ºC water bath for 60 min, to mimic the HT used above to trigger interstitial Dox release, and Dox release was imaged using a fluorescence imager (IVIS). **Figure 6** represents the relative increase in Dox fluorescence (to zero time point) in the euthanised and anaesthetised mice following 60 min HT. Our results showed a two-fold reduction in fluorescence intensity in the mice injected with the ultrafast-releasing LTSL-Dox formulation (**Figure 6A & 6C**). A minimum increase in the fluorescence was observed in the anaesthetised mice injected with TTSL-Dox or NTSL-Dox combined with 60 min HT (**Figure 6B**). On the other hand, and as anticipated, stopping the blood flow in the tumour in the euthanised mice, significantly increased the Dox fluorescence in the mice injected with LTSL-Dox and TTSL-Dox compared to NTSL-Dox. Dox release from LTSL-Dox and TTSL-Dox, resulted in an 8-10 and 6-fold increase, respectively (**Figure 6B & 6D**), highlighting the critical role that tumour blood flow plays in drug clearance from heated tumours. However, further studies are warranted to quantify Dox clearance from in tumours with different blood flow rates.

## Discussion

HT has been combined with TSL to enhance drug bioavailability while reducing systemic toxicity. HT regimens have been manipulated to obtain intravascular or interstitial drug release. The intravascular drug release triggers Dox-release from circulating TSL in the tumour vasculature. This type of release has been adopted clinically 10. Such an approach can only be applied with drugs that are readily taken up by the tumour tissue and requires high heating precision to ensure only heating the tumour tissue. Moreover, the intravascular release requires fast content releasing TSL that, however, does display intrinsic instability and premature leakage in blood 8, 14, 17. In contrast to the intravascular drug release, which is still at the preclinical phase, interstitial drug release has been combined with serum-stable, long-circulating liposomes with an intermediate release profile to be released interstitially 11, 12. A new hybrid regimen has been introduced, which consists of a two-step HT approach to enhance drug delivery and release in tumours, where it is challenging to apply HT precisely 11. The latter approach is based on applying HT prior to TSL injection to benefit from HT's enhanced tumour accumulation caused by HT, followed by a second HT to trigger drug release from the extravasated TSL. Several studies have reported lower therapeutic efficacy of such an approach than mice injected with fast-releasing TSL-Dox formulations, where intravascular drug release occurs; however, limited investigations have been carried out to understand this finding 11-13. Li *et al.* previously showed that level of Dox in tumours treated with two-step HT was comparable to tumours treated with fast-releasing TSL combined with HT (intravascular release), but no evidence of *in vivo* Dox release was provided, which could have explained the lower therapeutic efficacy of two-step HT. Lokerse *et al*. studied the tumour accumulation of radiolabeled TSL using SPECT/CT in murine melanoma B16F10 and murine sarcoma BFS-1 tumour models following a two-step HT application. In that paper, the authors attributed the lower therapeutic efficacy of traditional TSL combined with two-step HT to a lower Dox level in tumours treated with fasting releasing TSL (intravascular release) due to Dox leakage from circulating TSL over time 13. It is worth mentioning that both studies used TTSL formulations slightly different to our TTSL and applied different 2-step HT regimens. This could add some discrepancy to our work, but the effect of second water bath HT could still be applicable, requiring further investigation.

Our previous publication evaluated the serums stability, blood circulation, organs distribution, tumour accumulation, and Dox release of dually labelled LTSL-Dox, TTSL-Dox and NTSL-Dox 14. Interestingly, TTSL-Dox exhibited long blood circulation with (10% of the injected dose was detectable in the blood up to 24 h) and showed high serum stability with more than 50% Dox was retained in TTSL-Dox after 24 h of injection. This significant amount of the encapsulated Dox could be released following the second HT application to enhance its efficacy. In the present work, we systematically studied, and for the first time, the effect of a single and two water bath HT applications on Dox release from TSL with fast and intermediate release profile *in vivo*. Furthermore, we assessed the impact of mild localised HT on Dox clearance from tumour tissues, which was further correlated to the therapeutic efficacy of different TSL combined with one or two HT in two tumour models.

HT application in the clinic has varied between 30-90 min (NCT02567383, NCT02353858). Celsion has completed a Phase 3 study of ThermoDox® with radiofrequency ablation (RFA) for the treatment of hepatocellular carcinoma (HCC) (NCT02112656), and Phase 1 clinical trial (TARDOX study) for ThermoDox® in combination with high focused intensity ultrasound (HIFU) against liver cancer (NCT02181075). New Phase 1 clinical studies of Thermodox and MR-HIFU for treatment of relapsed solid tumors or pancreatic cancer are ongoing (NCT04791228, NCT02536183, NCT04852367). Despite the fact that water bath HT has been applied preclinically or clinically as whole-body hyperthermia, it could have the potential to locally treat superficial tumours. Furthermore, it is essential to highlight that multiple heating sessions have not been used with the clinically studied ThermodDox®, due to its low stability and ultrafast release profile, resulting in no added benefit to a single HT. However, with the advances in TSL, stable formulations with intermediate release profiles could benefit more for the multiple heating sessions, using water bath or other modalities, which could be clinically explored in the future. Undoubtedly, the current findings offer a better understanding of multiple HT on drug release from TSL.

HT is known to increase blood flow to improve drug delivery to the tumour tissue 18, however, the same phenomenon should be taken into account once two HT sessions are applied to trigger drug release interstitially. An earlier study showed that heating tumours and surrounding skin to 43 °C for 60 min increased the blood flow by a factor of 2, which gradually returned to normal within 3 hr (18). More interestingly, the authors of that study observed the lowest level of cis-diamminedichoroplatinum II (CDDP) in a mammary carcinoma model, when the drug was systemically administered at the end of the HT rather than the beginning. The authors attributed this low tumour accumulation due to the wash-out effect, where increased blood flow did not allow adequate time for the drug to be taken up by the cancer cells. Another study by Song *et al.* reported a 1.5-fold increase in blood flow in a R3230 tumour model heated at 41 °C for 60 min. Furthermore, the model exhibited a 2.5-fold higher blood flow than the baseline, which lasted to 24 hr. Scrutinizing the literature, it was found that the effect of HT on tumour blood flow was highly dependent on the temperature used, length of the treatment, and tumour type 19-21. In addition to the effect of HT on increasing blood flow to the tumour and neighbouring tissues, an interesting study by Avery *et al.* was published, reporting an accelerated lymphatic drainage from B16F10 tumours implanted in C57BL16 footpads, which persisted to few hours following HT application 22. In our study, we believe that the reduction in the TTSL-Dox fluorescence intensity in mice (**Figure 4**), and the lower level of ^14^C-Dox in tumours injected with an intermediate releasing TTSL formulation combined with two HT sessions were attributed to the washing of the released Dox from the tumour tissues (**Figure 5**). This was indirectly confirmed when Dox fluorescence intensity significantly increased in the tumours of euthanised mice injected with TTSL-Dox (**Figure 6**). Li *et al.* previously did not report such reduction in Dox levels following the second HT. However, in that study, no Dox quantification prior to the second HT was carried out. Furthermore, the second HT was applied 2 hr after liposome injection, which means that Dox wash-out from the tumour could have been masked by the continuous extravasation of the circulating TSL-Dox to the tumour tissue 11.

Murine melanoma B16F10 tumour model is known to be highly vascularised and exhibits a highly permeable vasculature (23). A recent study by Lokerse *et al.* has been published, which examined the effect of HT on tumour blood vessels (13). Histological examination of B16F10 tumour model revealed structural abnormalities in the tumour vasculature, less cellular packaging, and almost completely absent of extracellular matrix. This could explain the high tumour accumulation of our TTSL-Dox and NTSL-Dox, 24 hr post-HT (**Figure 1**), and the significant reduction in ^14^C-Dox levels in tumours treated with TTSL-Dox and subjected to second HT for 60 min (**Figure 5**). Despite the lower vascularisation/permeability of the MDA-MB-435 tumour model, where lower liposome extravasation was observed 24 hr post HT, compared to the B16F10 model (**Figure 1**), a lower but significant Dox clearance was consistently observed after the second HT (**Figure 5**). This could be explained by the quick wash-out of small drug molecules from the tumour Interstitium, compared to nano-sized NTSL-Dox (**Figure 5**).

Earlier studies of NTSL-Dox (Doxil^®^) combined with HT reported a slight improvement in the NTSL-therapeutic efficacy due to increased liposome tumour accumulation 24, 25. Our results confirmed that the higher tumour accumulation in mice treated with TTSL-Dox and NTSL-Dox (**Figure 1**) did not translate into higher therapeutic efficacy and survival compared to LTSL-Dox treated mice (**Figure 2 & 3**). Previously, a comparable tumour growth inhibition was observed in mice treated with slow-releasing TSL (DPPC:DSPC:DSPE-PEG2000 55: 50:5) or NTSL using the two-step HT approach 11. Although no proof of *in vivo* Dox release was provided, the authors speculated that Dox release from TSL-Dox improved Dox bioavailability, yielding similar efficacy to NTSL-Dox, which is in agreement with our results. In contrast to the MDA-MB-435 model (**Figure 3**), Dox-LTSL was less effective in delaying tumour growth in the B16F10 tumour model, where the inhibition effect was transient (up to 14 days) before the tumour grew exponentially (**Figure 2**). The short effect of LTSL-Dox in the B16F10 melanoma tumour model could be explained by the limited activity of LTSL-Dox on the tumour vasculature. LTSL-Dox has been described as anti-vascular agent that shuts tumours' blood supply 26, wheres vascular endothelium damage is caused by direct exposure to high local concentrations of Dox following the intravascularly release in the heated tumours. Our B16F10 model is a highly vascularised and fast-growing tumour model with *in vitro* doubling time of 13-20 hr 27, 28. Thus, we believe using a single dose of Dox-LTSL and HT was not sufficient to stop the tumor's blood supply and completely kill these fast-growing tumour cells. In agreement with our results, Lokerse *et al.* reported limited efficacy of TSL-Dox combined with HT in the highly vascularised B16F10 tumour model 13. Furthermore, Dewhirst's group previously evaluated the therapeutic efficacy of LTSL-Dox in different human and murine tumour models using a clinically relevant HT protocol 26, where LTSL-Dox was the least effective in a murine fast-growing mouse mammary tumour model (4T07) with a doubling time of 14 hr. These findings highlight the key role that tumor type plays, beside the TSL characteristics and HT protocol in determining HT's overall therapeutic efficacy in combination with chemotherapy.

## Conclusion

Applying a second water bath HT session to release Dox from the extravasated TTSL (DPPC:HSPC:Chol:DSPE-PEG_2000_) was not therapeutically superior to a single HT application due to Dox clearance from the extravasated TTSL-Dox. Our findings revealed for the first time that enhanced tumour blood perfusion caused by the second water bath HT session (60 min) could be seen as a hurdle for TTSL-Dox's anticancer efficacy, which also could increase the systemic toxicity of the redistributed Dox to the blood circulation. Therefore, understanding the effect of multiple HT applications using water bath or other techniques on blood perfusion in different tumour models should be thoroughly assessed to decide on the efficacy and safety of the two-step or multiple HT combined with drug-loaded TSL. Moreover, alternative approaches, such high intensity focused ultrasound (HIFU), offering higher precision to trigger HT* in vivo* while minimising tumour wash-out, should be fully investigated 15, 16.

## Figures and Tables

**Table 1:** ** Physiochemical properties of all TSL-Dox used in the present study.** Lipid composition, size, surface charge and Dox encapsulation efficiency of TSL-Dox used in this study. The mean hydrodynamic diameter (nm), polydispersity index and surface charge of LTSL-Dox, TTSL-Dox, and NTSL-Dox were obtained using Zetasizer Nano ZS. (Malvern, UK). Dox encapsulation efficiency and TSL phase transition were determined using spectro-fluorometry, and DSC, respectively. (n = 3 ± SD)

Formulations	Lipid composition (molar ratio)	Size (nm) ± SD	PDI ± SD	ζ(mV) ± SD	% Encapsulation Efficiency ± SD	Phase transition (T_m_)
LTSL-Dox	DPPC:MSPC:DSPE-PEG_2000_ (86.6:9.6:3.8)	123 ± 11.0	0.06 ± 0.01	-11.1 ± 0.6	95 ± 6.5	41.5
TTSL-Dox	DPPC:HSPC:Chol:DSPE-PEG_2000_ (54:27:16:3)	118 ± 0.40	0.09 ± 0.02	-10.1 ± 0.5	92 ± 3.5	44.2
NTSL-Dox	HSPC:Chol:DSPE-PEG_2000_ (56.3:38.2:5.5)	114 ± 1.70	0.05 ± 0.01	-16.9 ± 1.3	93 ± 4.0	NA

**Table 2:** ** Mice median survival of all our therapy studies.** B16F10 tumour-bearing and MDA-MB-435 xenograft-bearing mice were injected intravenously with 5 mg Dox/kg of liposome-Dox formulations (LTSL-Dox, TTSL-Dox, NTSL-Dox) followed by 60 min HT by immersing the tumour-bearing leg into 43 °C water bath. Some mice were subjected to second HT (60 min) 24 hr post the first HT. Statistical analysis was performed using Prism software (** p < 0.01, *** p < 0.001). Statistical differences were compared to the control mice

Tumour model	1 HT				2 HT			
	Control	LTSL-Dox	TTSL-Dox	NTSL-Dox	Control	LTSL-Dox	TTSL-Dox	NTSL-Dox
B16F10	17	21 (** 0.0012)	21 (** 0.0029)	21 (** 0.0045)	17	21 (***0.0006)	21 (*** 0.0006)	23 (*** 0.0006)
MDA-MB-435	24	ND* 80% mice were alive by day 70) (**0.004)	32 (**0.005)	56.5 (*0.019)	27	ND* 70% mice were alive by day 70) (***0.0007)	36 (**0.005)	36 (**0.005)

*ND: not determined.
