# Supplementary material for: Mild hyperthermia accelerates doxorubicin clearance from tumour-extravasated temperature-sensitive liposomes
Source: Nanotheranostics. 2022 Jan 1;6(3):230–42. doi: 10.7150/ntno.61280 (PMC8824668; doi:10.7150/ntno.61280)
Supplement: Supplementary file 1 — Supplementary figures. [file ntnov06p0230s1.pdf]

Mild hyperthermia accelerates doxorubicin clearance from tumour-extravasated temperature-sensitive liposomes

Wafa' T. Al-Jamal[1,2*] and Kostas Kostarelos[1,3*]

[1]Nanomedicine Lab, UCL School of Pharmacy, University College London, Brunswick Square, London WC1N 1AX, UK.

[2] School of Pharmacy, Queen's University Belfast, United Kingdom

[3] Faculty of Medical &Human Sciences and National Graphene Institute, University of Manchester, Manchester, United Kingdom

To whom correspondence should be addressed:

Dr Wafa' T. Al-Jamal
School of Pharmacy
Queen's University Belfast
Belfast, BT9 7BL
United Kingdom
E-mail: w.al-jamal@qub.ac.uk

Supporting information

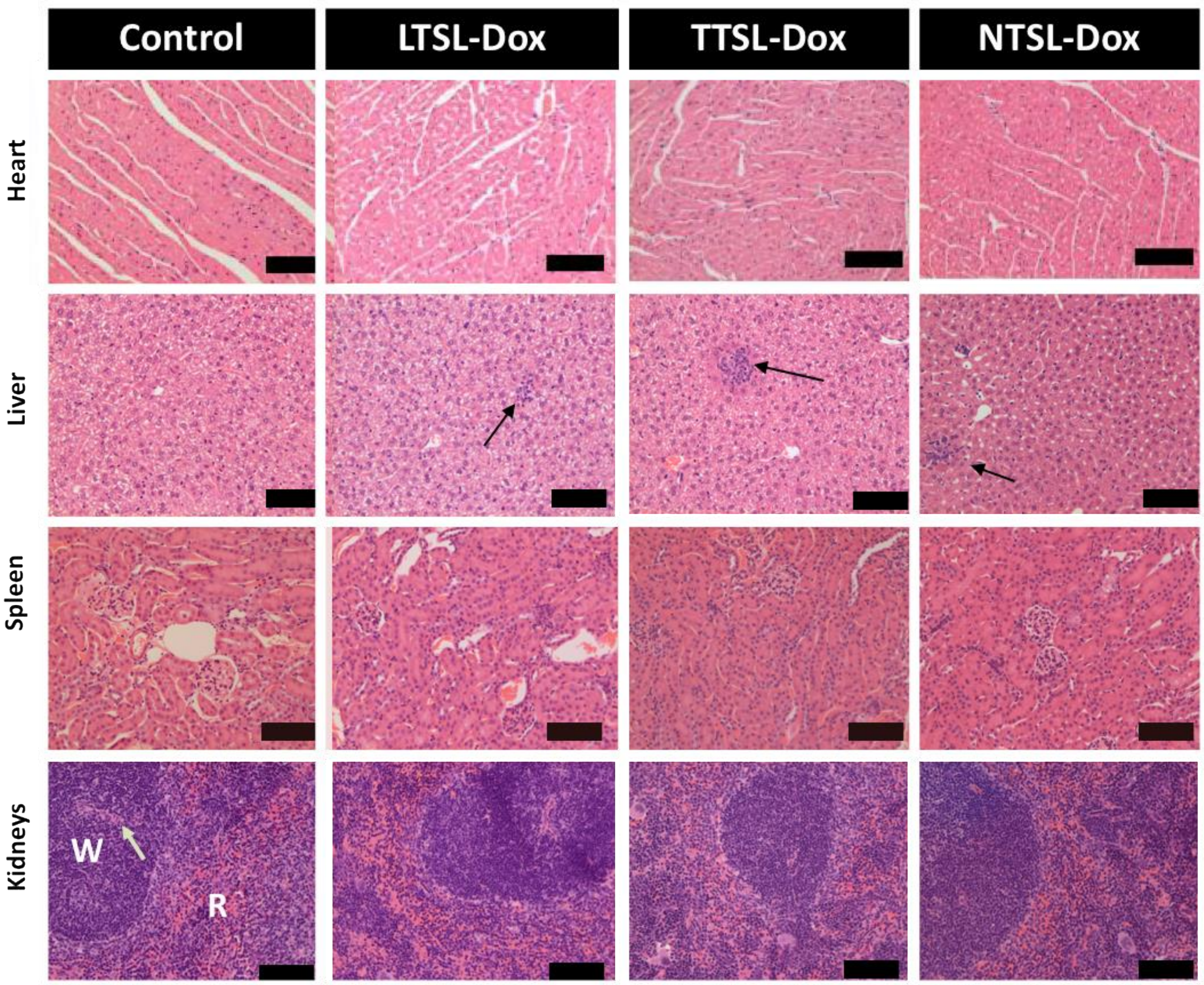

Figure S1: Histological examination of mice injected with TSL-Dox in combination with mild localised HT. H&E staining of heart, kidney, liver and spleen tissues from B16F10 tumour-bearing C57BL6 mice, three weeks following injection of 5 mg/kg Dox encapsulated in LTSL, TTSL, and NTSL. Magnification 10x and scale of images 10 µm (black bar). No histological abnormalities were observed in the tissues; expect some macrophages infiltration in the liver of the treated groups (as indicated by the black arrows).

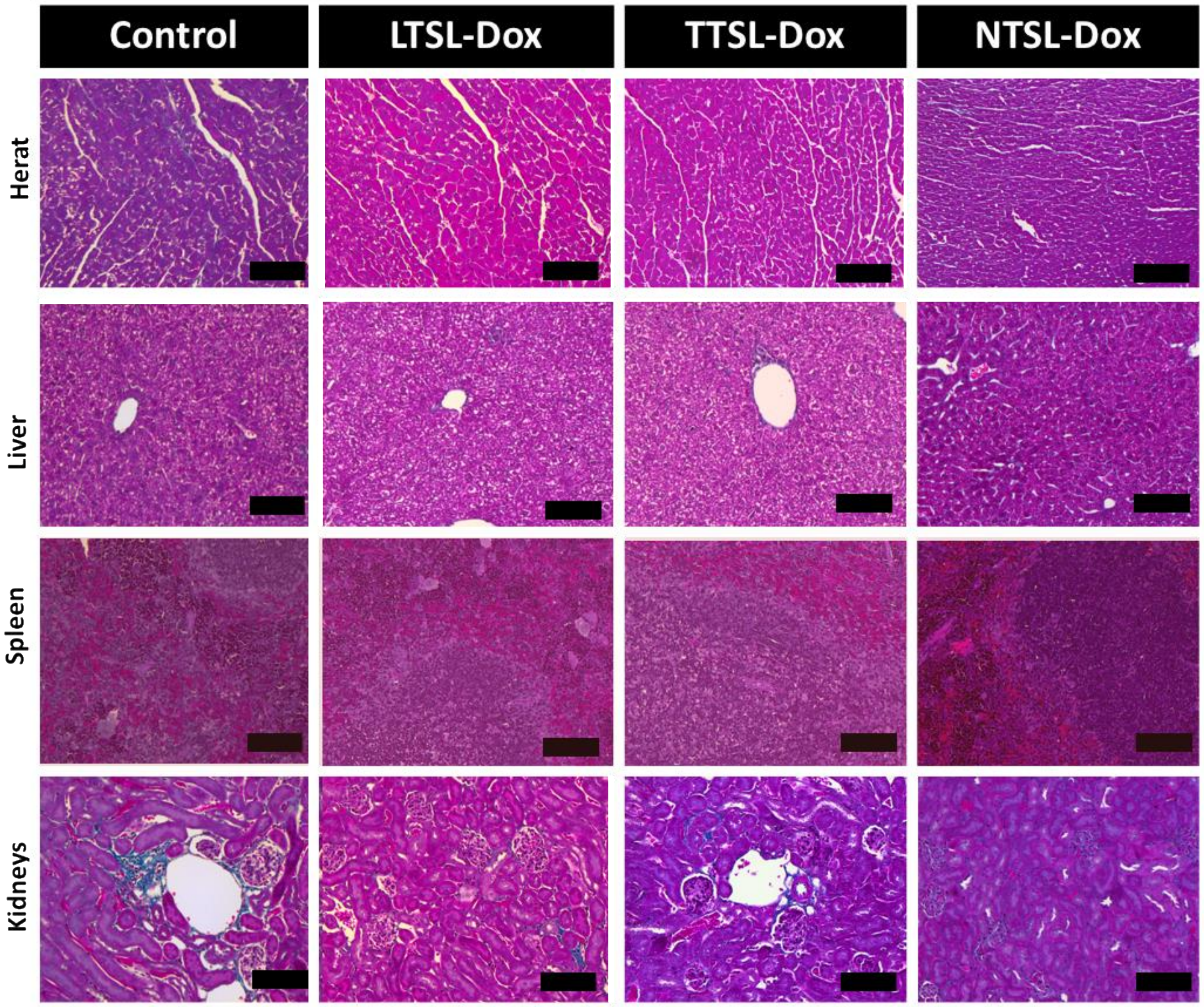

Figure S2: Histological examination of mice injected with TSL-Dox in combination with mild localised HT. Masson's Trichome staining of heart, kidney, liver and spleen tissues from B16F10 tumour-bearing C57BL6 mice, three weeks following injection of 5 mg/kg doxorubicin encapsulated in LTSL, TTSL, and NTSL. Magnification 10x and scale of images 10 µm (black bar). No signs of fibrosis was evident in all examined sections.

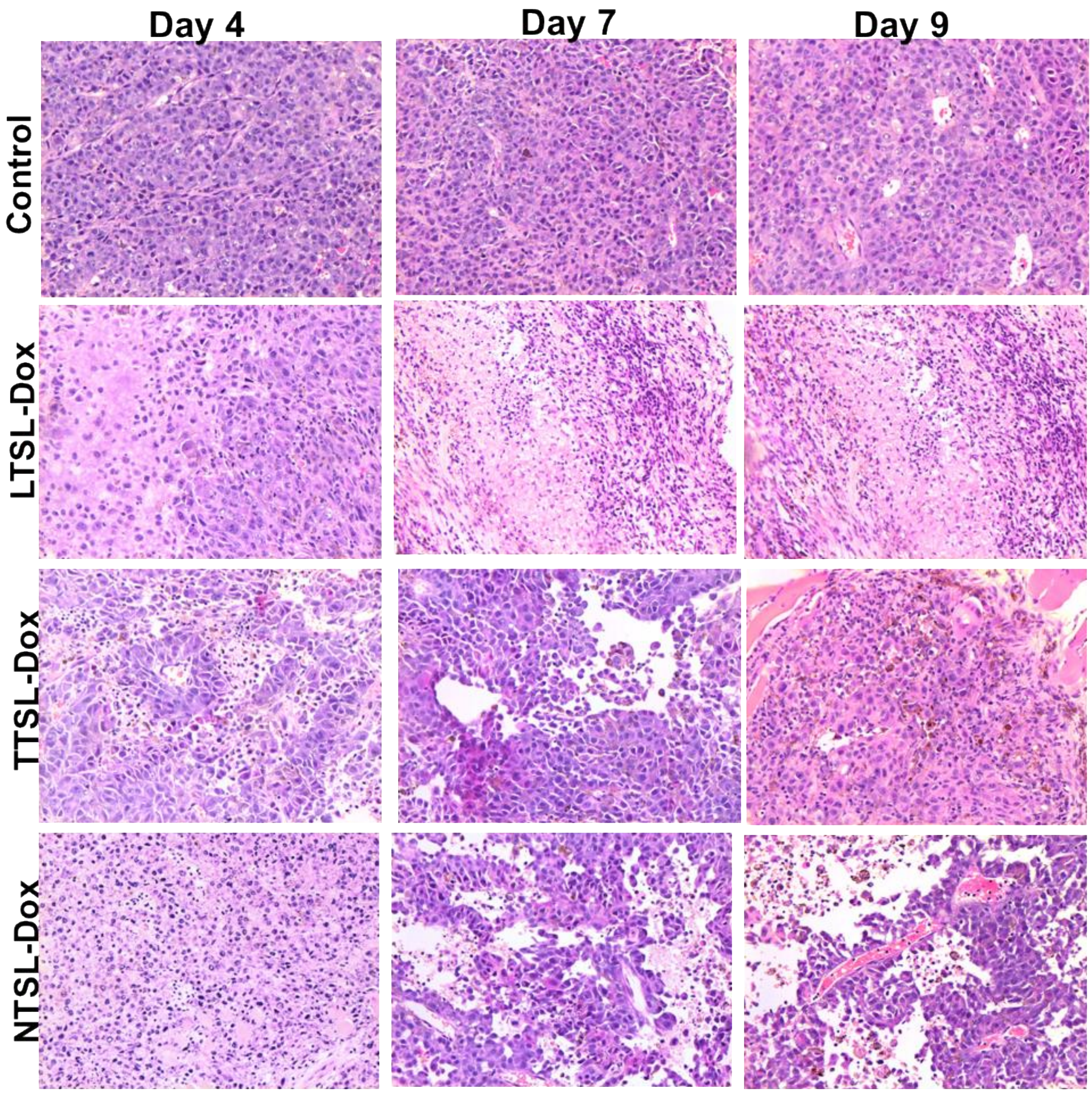

Figure S3: Histological examination of B16F10 tumours treated with TSL-Dox and a single HT session. H&E staining of B16F10 tumours injected intravenously with LTSL-Dox, TTSL-Dox, and NTSL-Dox at a dose of 5 mg/kg Dox in combination with 60 min mild localised HT session (Magnification 10x). Extensive tissue damage was observed in all treated groups (LTSL-Dox, TTSL-Dox, and NTSL-Dox) on day 4, 7 and 9 post the first HT treatment, compared to the control group.

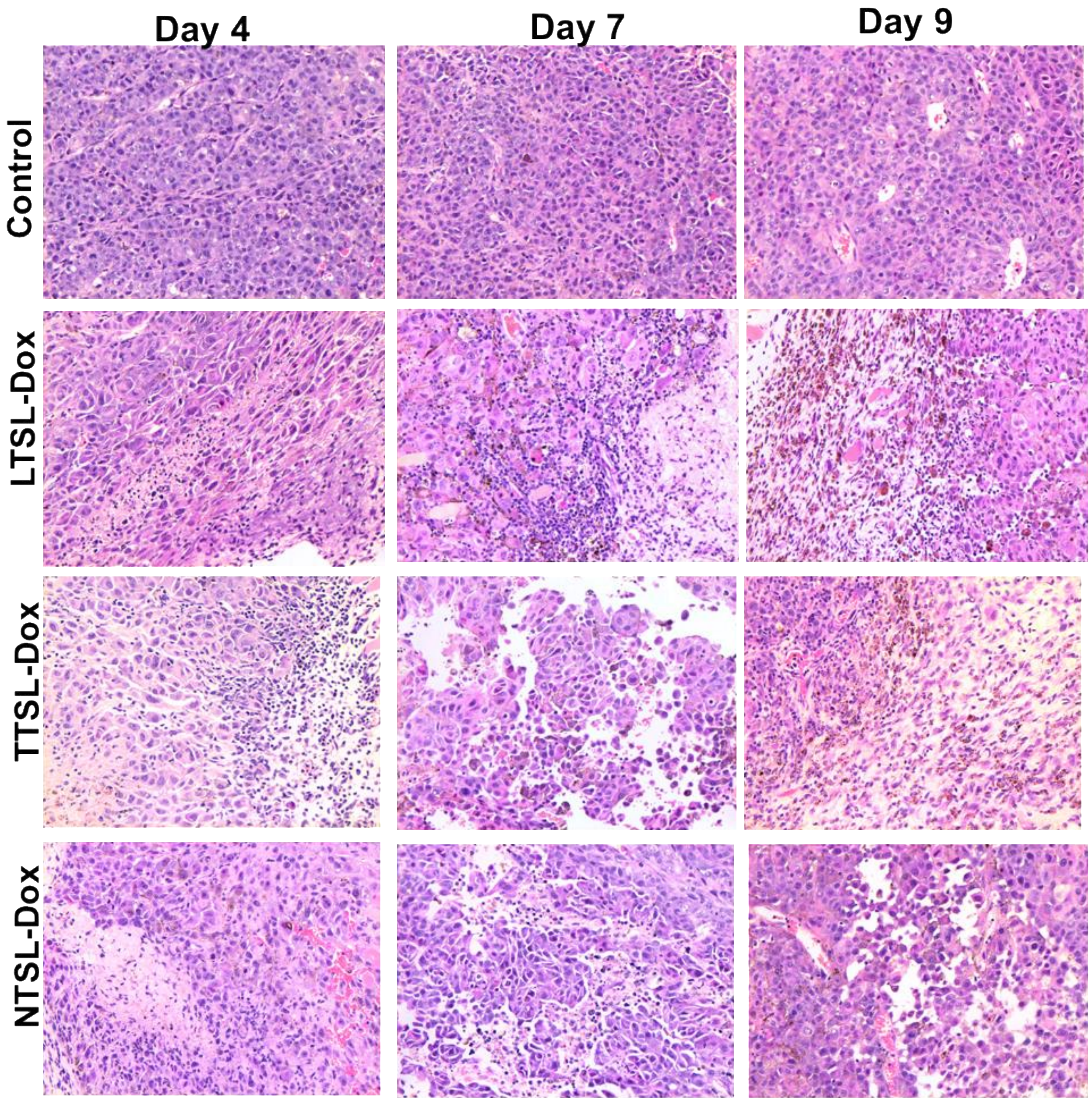

Figure S4: Histological examination of B16F10 tumours treated with TSL-Dox and two HT sessions. H&E staining of B16F10 tumours injected intravenously with LTSL-Dox, TTSL-Dox, and NTSL-Dox at a dose of 5 mg/kg Dox in combination with two HT sessions (immediately after injection and 24 hr post the first HT) (Magnification 10x). Extensive tissue damage was observed in all treated groups (LTSL-Dox, TTSL-Dox, and NTSL-Dox) on day 4,7 and 9 post first HT treatment, compared the control group.

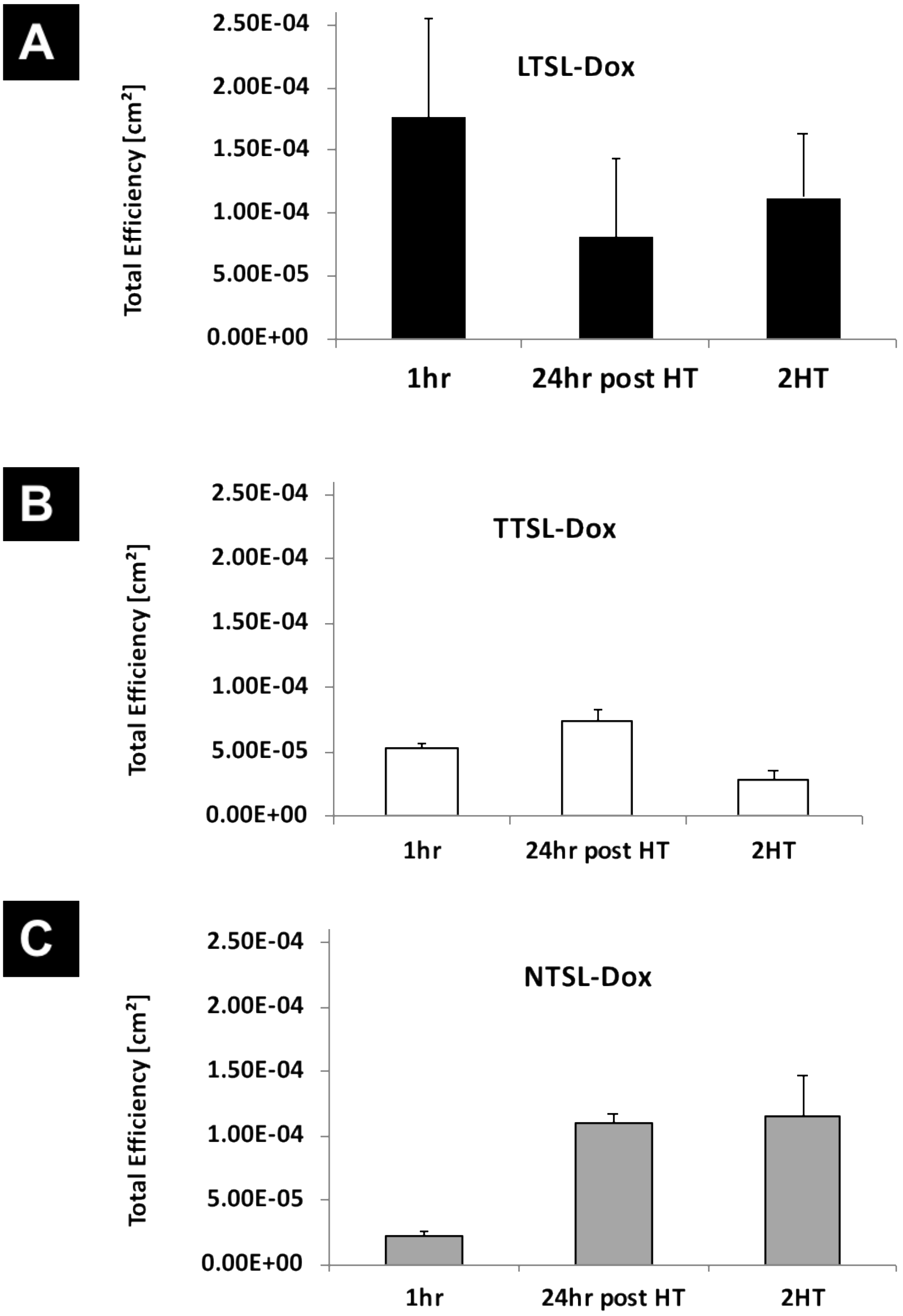

Figure S5: TSL-Dox release *in vivo.* TSL-Dox formulations were injected intravenously at 5 mg/kg Dox in human breast MDA-MB-435-xenograft bearing athymic nude mice. Live imaging of Dox release in human breast MDA-MB-435-xenograft bearing athymic nude mice, immediately after 1 hr HT, 24 hr post-HT and after the second HT treatment (2 HT). Dox release was assessed using the IVIS® Lumina II *In Vivo* Imaging System (Caliper Life Sciences Corp., Alameda, CA). Dox fluorescent intensity at the tumour site was quantified by drawing a region of interest (ROI) that covers the tumour area. Data are displayed in the unitless value of efficiency and represent the ratio of light emitted to light incident. Data are expressed as mean ± S.D (n=3)
